# Epidemic Cerebro-Spinal Meningitis in Missouri

**Published:** 1847-11

**Authors:** 


*Epidemic Cerebro-spinal Meningitis in Missouri*—In our number for August last, (p. 506,) we inserted in the "Record" department a communication, addressed to the *New Orleans Medical and Surgical Journal*, on the subject of a highly fatal disease, which prevailed in the early part of the year in Missisippi and Tennessee, and was manifestly an affection of the same character as one which visited with fearful malignity different towns of France, attacking principally the common soldiers of the garisons. It was seen also in Ireland in 1846; and everywhere has presented the same great general characters. The term *cerebro-spinal* seems to be applied to it with propriety, and it evidently attacks, in a marked manner, the gray matter in the centre of the cord—the nervous system of reflex actions. It would appear from the following extract of a letter, addressed to Professor Dunglison by Dr. W. C. Philips, dated Rocheport Boon county, Missouri, September 13th, 1847. that the scourge has been devastating localities widely apart from each other, and it is not improbable that it may extend elsewhere.

"I take the liberty," says Dr. Philips, in his intelligent epistle, "of advising you of the prevalence of a disease. that has excited considerable interest in this country, from its nondescript and fatal character.

"*Symptomatology*. There are no invariable symptoms attending it. Generally, the patient has been in ordinary health, pursuing his accustomed occupation, and the first indication he has of the approach of the disease is, perhaps, a pain in the head, foot, arms, legs, eye, brain, lungs, stomach, or bowels. Any one, or a number of these locations in connection, may be primarily affected—sometimes there is a sensation of cords drawing at the back of the neck, producing stiffness. In one case, there was contraction of the occipito-frontalis, and the muscles of the face. In many cases, there is so much soreness of the surface, as to render the smallest amount of clothing intolerable. It is usually ushered in with a chill of variable intensity, succeeded by similar reaction, and this reaction is followed in from 3 to 12 hours by a second chill, and if disorganization has not previously taken place, (which is often the case) it appears to accompany, or immediately succeed it. In many cases, there is excruciating pain in the arms, legs, and other parts of the body, and this pain, when located in the limbs, is often accompanied by swelling of the joints and more or less loss of the use of the limbs, with stiffness and inability to move them. These pains are sometimes permanent, but at others, shifting from one point to another. The neck is often drawn backwards or forwards at an angle of almost 45° from its natural position, and so rigidly fixed, that it would break it short off to force it to resume its physiological position, and is swollen very much larger than its ordinary size. The external surface is sometimes beautifully spotted; and in a few cases there was an exanthematous eruption. The nervous system is deeply involved. There may be profound coma,—wild and furious delirium,—subsultus tendinum, &c., apoplexy and paralysis. In one case, there was blindness of one eye, accompanied with permanent pain in the head; also swollen joints, (terminated fatally.) Lungs may or may not be implicated. Stomach the seat often of nausea and vomiting. Bowels, nothing characteristic. In a few cases, there was gastro-enteritis. Trismus occurred in one case. In other cases, there was an inability to swallow any thing, from the swollen and painful condition of the larynx and pharynx.

"*Etiology*. Most common from 10 to 15 years of age; but occurs at all periods of life. It resembles in many respects the prevailing disease of our country, (at the time of its occurrence last spring,) which was a modified form of pneumonia. There was, in both, the same soreness of the surface. The chill ushering in; the pneumonic symptoms and the swelling of the joints were similar; and the worst forms of the prevailing disease, and this malignant scourge, were similar in their duration, termination, &c. From these facts I infer, that they are produced by the same general cause, and that there exist local causes where it prevailed giving its malignant type. The disease is confined to a section of country in the Missouri River bottom, which was the seat of a great overflow, 1844, at which time an extensive layer of sand was deposited upon the soil, entombing large crops of vegetable matter. After the overflow, this bottom has been unusually healthy until last spring, whilst the rest of our country has suffered far more from disease than usual. Would it be philosophical or scientific to say this overflow produced these local causes?

"*Prognosis* is unfavorable: five-sixths die. In many cases, it is death *ab initio.* When fatal, it is generally so in from six hours to two days."—*Med. Examiner.*

